# Hung Out to Dry: Choice of Priority Ecoregions for Conserving Threatened Neotropical Anurans Depends on Life-History Traits

**DOI:** 10.1371/journal.pone.0002120

**Published:** 2008-05-07

**Authors:** Rafael Dias Loyola, Carlos Guilherme Becker, Umberto Kubota, Célio Fernando Baptista Haddad, Carlos Roberto Fonseca, Thomas Michael Lewinsohn

**Affiliations:** 1 Programa de Pós-graduação em Ecologia, Instituto de Biologia, Universidade Estadual de Campinas, Campinas, São Paulo, Brazil; 2 Departmento de Zoologia, Instituto de Biologia, Universidade Estadual de Campinas, Campinas, São Paulo, Brazil; 3 Departmento de Zoologia, Universidade Estadual Paulista Júlio de Mesquita Filho, Rio Claro, São Paulo, Brazil; 4 Universidade do Vale do Rio dos Sinos, São Leopoldo, Rio Grande do Sul, Brazil; University of California, Berkeley, United States of America

## Abstract

**Background:**

In the Neotropics, nearly 35% of amphibian species are threatened by habitat loss, habitat fragmentation, and habitat split; anuran species with different developmental modes respond to habitat disturbance in different ways. This entails broad-scale strategies for conserving biodiversity and advocates for the identification of high conservation-value regions that are significant in a global or continental context and that could underpin more detailed conservation assessments towards such areas.

**Methodology/Principal Findings:**

We identified key ecoregion sets for anuran conservation using an algorithm that favors complementarity (beta-diversity) among ecoregions. Using the WWF's Wildfinder database, which encompasses 700 threatened anuran species in 119 Neotropical ecoregions, we separated species into those with aquatic larvae (AL) or terrestrial development (TD), as this life-history trait affects their response to habitat disturbance. The conservation target of 100% of species representation was attained with a set of 66 ecoregions. Among these, 30 were classified as priority both for species with AL and TD, 26 were priority exclusively for species with AL, and 10 for species with TD only. Priority ecoregions for both developmental modes are concentrated in the Andes and in Mesoamerica. Ecoregions important for conserving species with AL are widely distributed across the Neotropics. When anuran life histories were ignored, species with AL were always underrepresented in priority sets.

**Conclusions/Significance:**

The inclusion of anuran developmental modes in prioritization analyses resulted in more comprehensive coverage of priority ecoregions–especially those essential for species that require an aquatic habitat for their reproduction–when compared to usual analyses that do not consider this life-history trait. This is the first appraisal of the most important regions for conservation of threatened Neotropical anurans. It is also a first endeavor including anuran life-history traits in priority area-selection for conservation, with a clear gain in comprehensiveness of the selection process.

## Introduction

Amphibian populations are declining worldwide and this is causing growing concern [1], [2]. As a group they are also extremely endangered. Of the 6,184 extant amphibian species [3], nearly one-third is globally threatened [4]. In the Neotropics, about 35% of anuran species were classified by The World Conservation Union (IUCN) as “critically endangered”, “endangered” or “vulnerable”. If we add species considered to be “near threatened” the percentage of threatened amphibians increases to 41%. Furthermore, relative to other animal groups, an outstandingly high proportion of amphibians are in higher threat categories [4]. These high threats at the population and species level demand effective strategies to devise conservation efforts for amphibians worldwide.

Among the leading factors that threaten amphibians, habitat loss, habitat fragmentation, and habitat split are the most important and, perhaps, the major causes of species' extinction in general [1], [4]–[6]. Recently, many studies have focused on the widespread distribution of chytridiomycosis (an infection caused by the fungus *Batrachochytrium dendrobatidis*), currently considered to be the main cause of amphibian population declines in undisturbed areas [2], [5], [7]–[9]. In these studies, the pathogen primarily affected species with an aquatic larval stage such as stream- and pond-breeders, whereas most species with terrestrial development (i.e., species whose development can be completed outside water bodies) were less affected.

Anuran species with different developmental modes of reproduction respond to habitat disturbance in different ways [6], [10]–[13]. Species with aquatic larvae are expected to suffer mainly with habitat split, as the disconnection between suitable aquatic and terrestrial habitats forces this group to perform compulsory breeding migrations through unfamiliar hostile habitats [6]. On the other hand, species with terrestrial development are expected to suffer mainly with habitat loss and fragmentation, as their life cycle depends particularly on the integrity and connection of vegetation remnants. Therefore, the effect of habitat changes on species with different developmental modes depends on their particular life-history traits, such as migration patterns, habitat use and ability to cope with biotic and abiotic microhabitat changes caused by disturbances [6], [14], [15]. For this reason, species with different life-history traits require distinct conservation strategies to be effectively protected, and therefore, the inclusion of ecological traits (e.g. reproductive modes, extinction risk) in conservation assessments and planning helps to improve reserve networks and to increase the effectiveness of proposed priority sets see [16].

Insufficient information for targeting conservation efforts is a major obstacle to the conservation of tropical biodiversity [17], [18]. As a result, the initial goal of large-scale strategies for conserving biodiversity is to identify regions of high conservation value that are significant in a global or continental context and then direct more detailed conservation assessments towards such areas [19], [20]. The most important criterion for locating and designing reserve systems should be to achieve maximum representation of biodiversity with the smallest possible cost [21], [22]. Several algorithms have been developed to create a reserve system that maximizes the representation of biodiversity in a region see [23]. Currently, one of the most efficient ways to decide which set of areas comprises the most inclusive representation of species for a particular region is through interactive site-selection heuristic or optimal algorithms based on complementarity [24]–[27].

In this paper we used the WWF's Wildfinder database [28], which encompasses 700 threatened anuran species in the 119 Neotropical Ecoregions, to identify minimum ecoregion sets that should be sufficiently covered in a reserve system to represent all threatened Neotropical anurans of each developmental mode (i.e. the aquatic larvae species and the terrestrial development species). We also compared the effectiveness of priority sets in representing species of different developmental modes when species subsets are treated separately according to this life-history trait, and when they are all considered together. Finally, we discuss how the inclusion of species biological traits such as life-history traits can enhance prioritization exercises for biodiversity conservation.

## Results

### Patterns of species richness and irreplaceability

**Figure 1:**
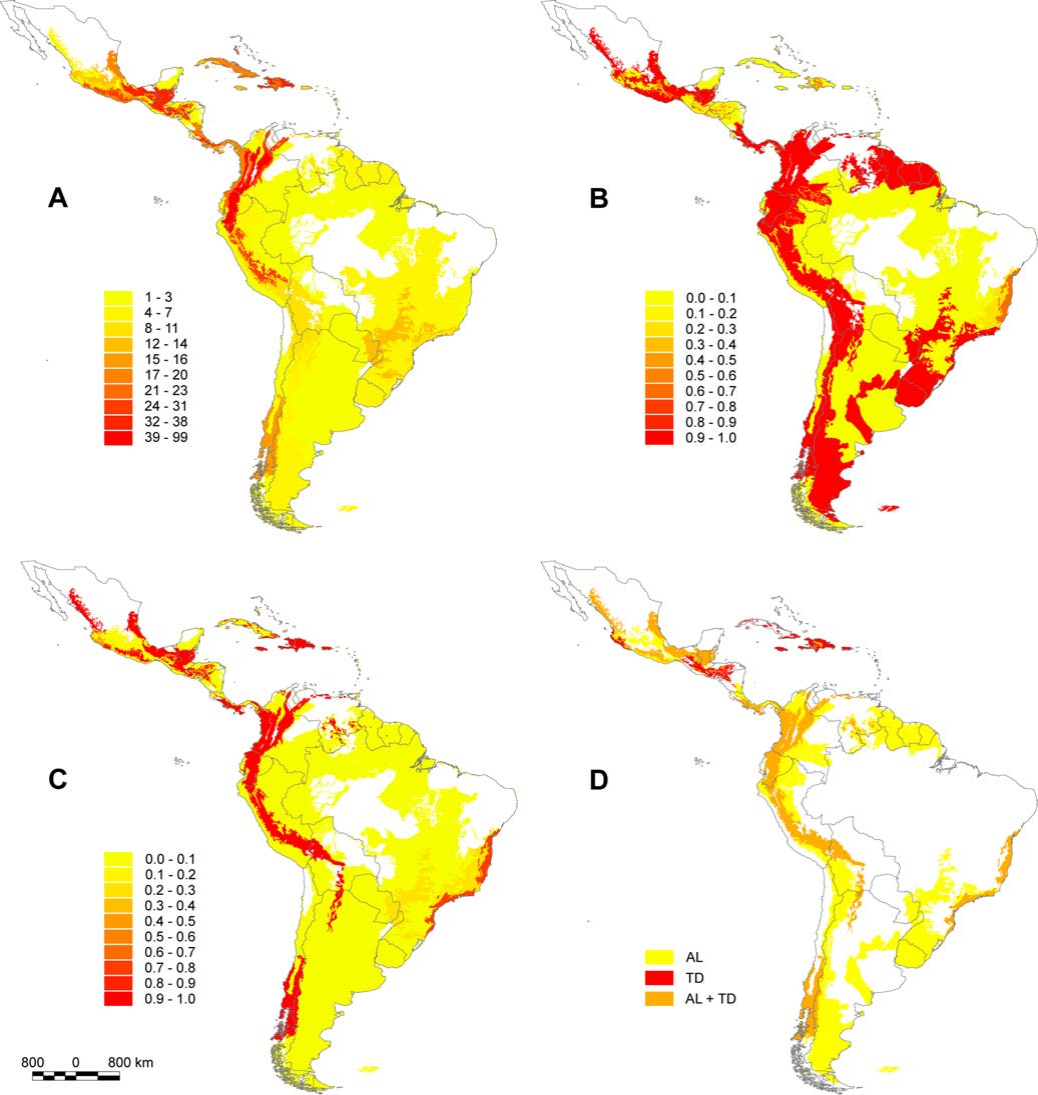
Pattern of species richness, irreplaceability and minimum ecoregion sets for representing threatened Neotropical anurans. Spatial patterns of threatened anuran species richness across Neotropical ecoregions (A) and spatial patterns of irreplaceability estimated by the frequency of ecoregions in the 100 optimal solutions obtained with all threatened anuran species with aquatic larvae (B) and terrestrial development (C) found in the Neotropics. Map showing minimum ecoregion sets (n = 66 ecoregions) required for representation of all threatened anuran species with different developmental modes (D), both those with aquatic larvae (AL = yellow, n = 26 ecoregions) and those with terrestrial development (TD = red, n = 10 ecoregions). Ecoregions of high importance for species of both developmental modes (AL+TD, n = 50 ecoregions) are represented in orange.

Threatened anuran species are concentrated in southern Mexico, the tropical Andes, and rainforests of Colombia and Venezuela (Figure 1A). Other ecoregions with high levels of species threat are found in the Caribbean Islands (Figure 1A).

We found that 50 ecoregions were included in all 100 optimal sets necessary to represent each species with aquatic larvae at least once (Figure 1B). These areas of high irreplaceability are concentrated in Mexico, Central America, the Tropical Andes, southern South America, and eastern Brazil (Figure 1B). Some ecoregions–such as the Atlantic moist forests from Brazil, other areas in Mexico and the Caribbean Islands–figured in at least 50% of all optimal sets (Figure 1B). On the other hand, only 34 ecoregions were included in all 100 optimal sets necessary to represent each species with terrestrial development at least once (Figure 1C). These ecoregions are located in Mexico, Costa Rica (the Talamancan montane forests), the Tropical Andes, Chile and Brazil (Figure 1C).

### Minimum sets of ecoregions for species representation in each developmental mode

**Figure 2:**
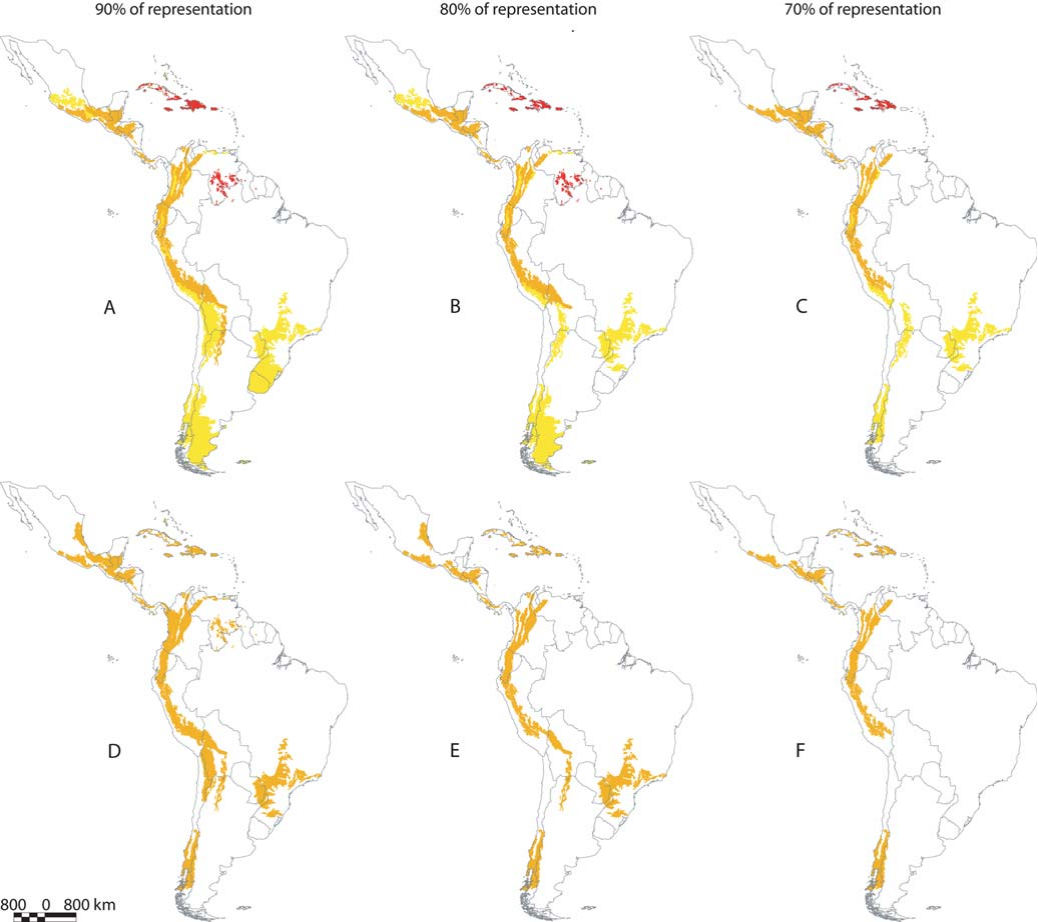
Key ecoregion sets for threatened Neotropical anurans obtained with or without discriminating species according to their developmental modes. (A–C) Maps showing the minimum ecoregion sets required for representation of species with different developmental modes, both those with aquatic larvae (AL = yellow) and those with terrestrial development (TD = red)-at different cutoff levels of species representation (95, 80, and 70%). Ecoregions of high priority for species of both developmental modes (AL+TD) are represented in orange. (E–G) Maps show minimum ecoregion sets required for representation of anuran species at different cutoff levels of species representation (95, 80, and 70%).

The application of the simulated-annealing algorithm on the species occurrence matrix revealed that a key ecoregion set of 66 ecoregions must be sufficiently covered in a reserve system, in order to represent all threatened anuran species in the Neotropics (Figure 1D, Table S1). Among these ecoregions, 30 were classified as priority for all species, 26 ecoregions were of high priority exclusively for species with aquatic larvae, and 10 ecoregions only for species with terrestrial development (Figure 1D, Table S1). The total amount of land area covered by our combined priority set spans almost 33% of the entire Neotropical region, of which *ca.* 22%, 1%, and 11% correspond to key ecoregion sets for species with aquatic larvae, terrestrial development or both developmental modes, respectively (Table S1). Key ecoregions for both developmental modes or only for terrestrial development species are highly concentrated in the Andes and more widespread across Mesoamerica (Figures 1D and 2A–C). Conversely, ecoregions particularly important for preserving threatened aquatic larvae species are widely distributed across the Neotropics, including important southern non-forest areas such as the Patagonian steppe and the Argentine Espinal (see Figures 1 and 2A–C).

Analyses that separated anurans according to their developmental modes resulted in more comprehensive priority sets (Figure 2); with more species represented from either group (Table 1). Species with aquatic larvae are increasingly underrepresented when conservation targets are progressively lowered from 95 to 70% in analyses that do not discriminate developmental modes; moreover, species with aquatic larvae never attain the intended conservation target, and ecoregions excluded from priority sets were mainly those important for this species group (Tables 1 and S2; Figure 2D–F). When analyzed separately, the percentage of species with aquatic larvae represented is closer to those with terrestrial development, though always lower than the latter (Table 1; Figure 2D–F).

**Table 1:** Representation of threatened Neotropical anurans in priority sets of ecoregions attained under different conservation targets.

Conservation target	Without discriminating anuran developmental modes			Discriminating anuran developmental modes		
	Number of ecoregions	AL	TD	Number of ecoregions	AL	TD
95% of representation	37	**91%**	98%	44	95%	97%
90% of representation	29	**84%**	96%	36	91%	97%
80% of representation	20	**74%**	87%	25	82%	89%
70% of representation	13	**61%**	77%	17	71%	81%

Number of ecoregions included in priority sets and percentage of representation of threatened Neotropical anuran species with different developmental modes attained in priority ecoregion-setting exercises, when species were discriminated according to this life-history trait (right columns) or not (left columns). Rows show progressively decreasing conservation targets. AL = species with aquatic larvae; TD = species with terrestrial development. Bold numbers show instances where the intended conservation target is not attained.

**Figure 3:**
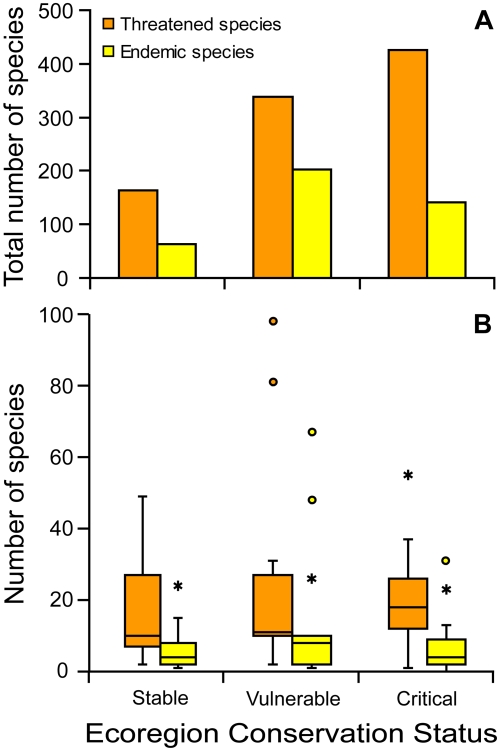
Conservation status of key ecoregions for the conservation of threatened Neotropical anurans. (A) Numbers of endemic and threatened species of Neotropical anurans found in ecoregions classified as Stable/Intact, Vulnerable or Critical/Endangered, according to [28]. (B) Distribution of the number of species found in ecoregions classified as Stable/Intact, Vulnerable or Critical/Endangered, according to [28]. Box plots indicate the range of the data between brackets, the middle two quartiles within the box, the median value as the midline, outside (_*_) and far outside (_°_) values.

Priority ecoregions with conservation status defined as “critical/endangered” harbor the majority of threatened Neotropical anurans; however, threatened species which are endemic to a given ecoregion are mostly found in “vulnerable” ecoregions (Figure 3A, Table S1). Stable and vulnerable ecoregions have also greater variation in the number of threatened species when compared with critical ones (Figure 3B, Table S1).

## Discussion

Optimal complementarity solutions based on biodiversity analyses have been successful in defining worldwide conservation networks [29], including those for anuran species [30]. Our analyses show that conservation efforts for threatened anurans in the Neotropics should be concentrated in a key set of 66 ecoregions, if all species with aquatic larvae or terrestrial development are meant to be represented. Patterns of geographic distribution of all amphibian species are not necessarily congruent with the distribution of threatened amphibian species [31]; hence our analysis cannot predict how effective the present priority sets will be in representing non-threatened anurans. This issue, although undoubtedly relevant, is beyond the scope of this paper–even though areas highlighted in this study are among the top β-diversity areas for amphibians in the Western Hemisphere [32].

Currently, most priority-setting assessments employ equal-area grids, and a number of effective tools have been developed for that purpose. These procedures are especially useful at smaller spatial scales, since they require a high density and coverage of records across grid units [33]. However, species records in the Neotropical region are fairly sparse and highly uneven, so that common grid-based analyses are less effective at the continental scale [34]. To a certain extent, the lack of field records may be overcome by summing expected distributions of species obtained through modeling [35]. Here, we chose to use ecoregions because these broad areas are defined according to physiographic and biotic features, and therefore should reflect zoogeographic boundaries more closely. They are also less sensitive to heterogeneity in distribution data than grid-based analyses [33] and are gaining support of major conservation organizations as well as of many government agencies (see also Materials and Methods).

The incorporation of developmental modes improved the comprehensiveness of minimum ecoregion sets. The strong species turnover in the Andes and Mesoamerica is primarily related to their high habitat heterogeneity, corresponding to an exceptional topographic variability found in these regions [32]. This favored the representation of Andean and Mesoamerican ecoregions; since our algorithm is based on complementarity, ecoregions that share few species will always be more complementary [25]. In fact, the complex topography and variety of environments mostly resulting from early tectonic events and climatic fluctuations in the Pleistocene and continuing to the present provide an array of habitats for an Andean herpetofauna that is more diverse than one might expect [36]. These geomorphological events probably are also responsible for generating high vertebrate β-diversity among ecoregions in Brazil [18], which harbors the richest amphibian fauna in the Neotropics [37].

Although the topographic history accounts for our priority set configuration, the high representation of threatened anurans in these regions can be further explained by other ecological phenomena. Wavy relief areas prevalent in Andean ecoregions have topographic features that favor the spatial separation between water sources and the remnants of natural vegetation cover. Natural remnants usually are concentrated in areas less suitable for agriculture, such as steeper slopes and hilltops [38], [39]. Anuran life-history traits entails not only particular habitat requirements, but also influences the landscape habitat use by each group, making species with aquatic larvae more liable to disappear from ecoregions whose terrestrial and aquatic breeding sites are more disjunct [6], [40]–[42]. It may be no coincidence that we observed higher counts of declining and threatened amphibians in these ecoregions [8], where the enforcement of laws that protect riparian vegetation thus becomes especially critical. Furthermore, high infection rates by chytridiomycosis in many Andean and Mesoamerican areas relatively protected from human influence strongly contribute to such a pattern [2], [43]. Another factor which may account for this pattern is the distinct historical dispersal of anurans with aquatic larvae or terrestrial development [8], [9], [13]. Species with aquatic larvae disperse mainly through riverflows. Hence, these species could become widespread across many areas, suffering fewer chorographic restrictions than species with terrestrial development, which should tend to be confined in certain sites, increasing β-diversity at a regional scale. If so, this could also explain why Andean ecoregions, along with those found in tropical forests of Mesoamerica, were highly represented in our priority sets, and reinforces the separation of anurans according to their developmental modes [6], [44]. Note, however, that geographic range (expressed as number of ecoregions) is not significantly different between species with aquatic larvae and terrestrial development.

Our priority sets are congruent with important areas indicated for the conservation of amphibians, as well as other vertebrates, derived from regional [45]–[47] and continental studies [5], [32], [48], [49]. Such congruence is especially high in the Andes and in Mesoamerica, where altitudinal range seems to play the most important role in driving high levels of amphibian species richness, endemism and threat [32], [47]. Our results suggest that, for the most part, ecoregions valuable for conserving species with terrestrial development have experienced severe habitat reduction, mainly driven by livestock grazing and agricultural expansion [28]. On the other hand, the priority set for conserving species with aquatic larvae includes ecoregions whose water sources are severely impacted (e.g. large parts of the Andes, Central America, and some dry lands [28]). These ecoregions have lost their natural habitats especially in the most accessible and irrigated areas for agriculture, whereas drier ecoregions, such as savannas and open formations, are threatened by the introduction of exotic species and agriculture expansion, especially along rivers [28].

### Conclusions

To sum up, our results highlight sets of areas of particular interest for the conservation of threatened Neotropical anurans. The inclusion of anuran developmental modes in prioritization analyses resulted in a more comprehensive coverage of priority ecoregions–especially those essential for species that require an aquatic habitat for their reproduction–when compared to usual analyses that do not factor in life-history traits. Moreover, if such life-history traits are not taken into consideration, priority area-setting exercises tend to favor species with terrestrial development. This result is particularly important because several recent reports of population declines worldwide pointed to higher suppression rates in populations of species with aquatic larvae [6], [8], [9], [44]. We propose that, whenever feasible, conservation assessments should include key life-history traits in order to improve reserve networks and thus to increase the effectiveness of proposed priority sets see [16]. Because areas differ in quality, identification of a comprehensive set of natural areas, as presented here, is a first step towards an *in-situ* biodiversity maintenance strategy, which only subtends a much more complex process of policy negotiation and implementation. Complementarity among ecoregions will be especially instrumental in making complex judgments about trade-offs between diversity and redundancy at the anuran species level.

## Materials and Methods

### Study site

We focused our analyses to all the 119 terrestrial ecoregions of the Neotropics because it harbors a highly diverse amphibian fauna, representing half of the world's total species richness [5], and is one of the tropical regions in which amphibian population declines and species extinction are extremely elevated [4], [5], [44]. Although there are several classifications of Latin America biogeographical regions, we follow the WWF hierarchical classification of ecoregions [28], [50]. Conservation assessments within the framework of larger biogeographical units are gaining support of major conservation organizations as well as of many government agencies see [50]. Given that most conservation decisions and policies have to be met within national boundaries, ecoregions may correspond roughly to the largest operational units at which decisions can actually be taken and implemented [18], although the implementation of Conservation Area Network must be produced at smaller spatial scales such as State or Municipality.

### Data

The database used for the analyses contains the current species list of 1,970 anurans in the 179 Neotropical ecoregions [28]. We tallied the presence or absence of 700 threatened anuran species which occur in 119 terrestrial ecoregions of the Neotropics. Threatened species were those classified by the 2006 IUCN Red List as “critically endangered”, “endangered” or “vulnerable”. We had to exclude 208 threatened species from the analyses because they were not assigned to ecoregions in the available database. Information on updates, detailed descriptions of the process, and complete lists of sources can be obtained from the Web site indicated by [28]. Note that these datasets are periodically updated, and the files used in our analyses may differ from the most recent versions available from [4], [28]. We focused our analyses on threatened Neotropical anurans. The number of species in this vertebrate group is not static, as new species continue to be discovered [37], [51]. However, the areas from which species are most often described tend to be the same and will likely accentuate the patterns we present [51]. Systematic bias in the data may arise from differences in sampling efforts, as the distribution of amphibians or geographic areas (e.g. Central American ecoregions) for which sampling efforts have been more intense will be more reliable than those that are undersampled. As a safety measure against such biases, we excluded from the analyses anuran species with an IUCN Red List category of “data deficient” [4] because of the unreliability of their range maps, and therefore, their occurrence in the studied ecoregions.

### Analyses

In order to identify key ecoregion sets for anuran conservation, we grouped species by their developmental mode, either with aquatic larvae (n = 336 species) or terrestrial development (n = 364 species). The determination of each developmental mode was based on the 31 reproductive modes of Neotropical anurans recognized by [52]. Species with reproductive modes that do not require aquatic habitats for their development were classified as species with terrestrial development, whereas species that do require an aquatic habitat for larval development were classified as species with aquatic larvae.

We used an optimization procedure to select the minimum number of ecoregions necessary to represent all species at least once, based on the complementarity concept [24]–[27]. For each anuran subset (i.e. species with aquatic larvae or terrestrial development), we ran a simulated annealing procedure in the Site Selection Mode (SSM) routine of the SITES software program [53]–[54] to find these combinations of ecoregions. We set the analyses parameters to 100 runs and 20 million iterations. We also set a relatively high penalty value for losing a species, so that every solution represented all species with a minimum number of ecoregions. Because there are frequently multiple combinations of ecoregions that satisfy this representation goal in each conservation scenario, we combined alternative solutions into a map in which the relative importance of each ecoregion is indicated by its rate of recurrence in optimal subsets (see Fig. 1B–C). This is also an estimate of the irreplaceability of ecoregions [55], ranging from 0.0 (minimum irreplaceability) to 1.0 (maximum irreplaceability) see [56].

This algorithm represents one possible solution to a problem known as the reserve site selection problem [29], which can be represented formally as follows: maximize
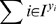
(1)subject to
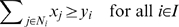
(2)

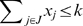
(3)

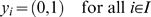
(4)

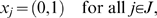
(5)where *J* = {*j*|*j* = 1, …, n} denotes the index set of candidate ecoregions from which to select, and *I* = {*i*|*i* = 1, …, m} denotes the set of the species to be covered. The set *N_i_*, a subset of *J*, is the set of candidate ecoregions that contain species *i*. The variable *x_j_* = 1 if ecoregion *j* is selected, 0 if ecoregion *j* is not selected. Constraint (3) limits the total number of ecoregions selected to no more than *k*. The variable *y_i_* will be 1 except when *x_j_* = 0 for all *j* in *N_i_* (since constraint (2) will force *y_i_* = 0 in that case)–i.e., constraint (2) enforces that the species not be counted as preserved if none of its ecoregions is selected [29].

The algorithm we used–which is driven by patterns of β-diversity–has been considered one of the most efficient approaches to define priority area sets for species conservation [24]–[27], [29], because including patterns of β-diversity in area selection algorithms captures variation in species communities, helping to maintain ecological and evolutionary processes in addition to underlying environmental heterogeneity necessary for long-standing persistence [32].

Ecoregions highlighted in our analyses were designated as the highest priority set. Minimum sets obtained from these analyses were drawn on a map of Neotropical ecoregions, as defined by [50], using ArcView GIS 3.2 (ESRI, Redmond, California). Shapefiles and associated attribute tables were obtained from [28]. Maps were combined to reveal the minimum set of ecoregions that should be included in a reserve system in order to represent all of anurans with aquatic larvae and of those with terrestrial development. We employed an equal-area cylindrical projection in all maps.

Finally, we compared the total coverage of species with aquatic larvae or terrestrial development in priority sets produced with different conservation targets (95, 90, 80 and 70% of threatened anuran representation). The analyses were repeated with and without discrimination for anuran developmental modes. Maps showing the minimum set of ecoregions obtained in each of these conservation targets were also produced as described above.

## Supporting Information

Table S1 Priority ecoregion sets for threatened Neotropical anurans with terrestrial development and aquatic larvae. Key ecoregion set (n = 66) proposed for representing all threatened Neotropical anuran species with different developmental modes (AL = aquatic larvae, TD = terrestrial development). Numbers in parentheses represent endemic species. Ecoregion conservation status obtained from [28]; threatened species combine those classified in the 2006 IUCN Red List as critically endangered, endangered or vulnerable.
(0.15 MB DOC)

Table S2 Priority ecoregions included (indicated by x) in priority sets attained with or without discriminating anuran developmental modes under different targets of species representation (90, 80 and 70%). For threatened species richness, numbers in parentheses represent endemic species. Threatened species combine those classified in the IUCN 2006 Red List as critically endangered, endangered or vulnerable.
(0.12 MB DOC)
