# Supplementary material for: Hung Out to Dry: Choice of Priority Ecoregions for Conserving Threatened Neotropical Anurans Depends on Life-History Traits
Source: PLoS One. 2008 May 7;3(5):e2120. doi: 10.1371/journal.pone.0002120 (PMC2361192; doi:10.1371/journal.pone.0002120)
Supplement: Table S1 — Priority ecoregion sets for threatened Neotropical anurans with terrestrial development and aquatic larvae. Key ecoregion set (n = 66) proposed for representing all threatened Neotropical anuran species with different developmental modes (AL = aquatic larvae, TD = terrestrial development). Numbers in parentheses represent endemic species. Ecoregion conservation status obtained from [28]; threatened species combine those classified in the 2006 IUCN Red List as critically endangered, endangered or vulnerable. (0.15 MB DOC) [file pone.0002120.s001.doc]

Table S1. Priority ecoregion sets for threatened Neotropical anurans with terrestrial development and aquatic larvae.

| **Ecoregion code** | **Ecoregion name** | **Conservation status** | **Priority** | **Threatened species richness** | | **Area (km^2^)** |
| --- | --- | --- | --- | --- | --- | --- |
|  |  |  |  | **AL** | **TD** |  |
| NT0103 | Bahia coastal forests | Critical | TD, AL | 86 (0) | 15 (0) | 109700 |
| NT0105 | Bolivian Yungas | Vulnerable | TD, AL | 4 (4) | 7 (6) | 90500 |
| NT0109 | Cauca Valley montane forests | Critical | TD, AL | 10 (2) | 12 (7) | 32100 |
| NT0114 | Chimalapas montane forests | Intact | TD, AL | 12 (1) | 5 (1) | 2100 |
| NT0115 | Chocó Darién moist forests | Intact | TD, AL | 6 (2) | 13 (2) | 73600 |
| NT0117 | Cordillera La Costa montane forests | Vulnerable | TD, AL | 3 (1) | 8 (7) | 14300 |
| NT0118 | Cordillera Oriental montane forests | Vulnerable | TD, AL | 15 (4) | 12 (4) | 67900 |
| NT0121 | Eastern Cordillera real montane forests | Vulnerable | TD, AL | 51 (31) | 30 (17) | 102500 |
| NT0130 | Isthmian-Pacific moist forests | Critical | TD, AL | 6 (1) | 10 (1) | 29300 |
| NT0136 | Magdalena Valley montane forests | Critical | TD, AL | 31 (14) | 24 (17) | 105100 |
| NT0145 | Northwestern Andean montane forests | Vulnerable | TD, AL | 63 (48) | 35 (19) | 81200 |
| NT0153 | Peruvian Yungas | Critical | TD, AL | 10 (7) | 20 (16) | 186700 |
| NT0154 | Petén-Veracruz moist forests | Critical | TD, AL | 13 (2) | 20 (2) | 149100 |
| NT0159 | Santa Marta montane forests | Vulnerable | TD, AL | 4 (3) | 7 (5) | 4800 |
| NT0160 | Serra do Mar coastal forests | Critical | TD, AL | 195 (3) | 32 (1) | 104800 |
| NT0161 | Sierra de los Tuxtlas | Critical | TD, AL | 4 (1) | 5 (3) | 3900 |
| NT0165 | Southern Andean Yungas | Vulnerable | TD, AL | 3 (2) | 8 (2) | 61100 |
| NT0167 | Talamancán montane forests | Intact | TD, AL | 13 (4) | 25 (11) | 16300 |
| NT0169 | Pantepuis | Intact | TD, AL | 8 (7) | 2 (1) | 48800 |
| NT0175 | Venezuelan Andes montane forests | Vulnerable | TD, AL | 7 (6) | 24 (20) | 29400 |
| NT0176 | Veracruz moist forests | Critical | TD, AL | 8 (1) | 10 (2) | 69100 |
| NT0178 | Western Ecuador moist forests | Critical | TD, AL | 13 (2) | 9 (1) | 34100 |
| NT0220 | Lesser Antillean dry forests | Critical | TD, AL | 3 (0) | 3 (0) | 100 |
| NT0228 | Sinaloan dry forests | Critical | TD, AL | 27 (0) | 10 (1) | 77500 |
| NT0305 | Hispaniolan pine forests | Critical | TD, AL | 22 (6) | 1 (0) | 11600 |
| NT0309 | Sierra Madre del Sur pine-oak forests | Critical | TD, AL | 8 (1) | 18 (8) | 61200 |
| NT0404 | Valdivian temperate forests | Critical | TD, AL | 2 (1) | 14 (12) | 248100 |
| NT1003 | Central Andean wet puna | Vulnerable | TD, AL | 4 (3) | 6 (5) | 117300 |
| NT1004 | Cordillera Central páramo | Intact | TD, AL | 2 (1) | 5 (3) | 12200 |
| NT1006 | Northern Andean páramo | Intact | TD, AL | 35 (17) | 14 (7) | 30000 |
| NT0120 | Cuban moist forests | Vulnerable | TD | 23 (2) | 0 (0) | 21400 |
| NT0127 | Hispaniolan moist forests | Critical | TD | 36 (13) | 1 (0) | 46000 |
| NT0131 | Jamaican moist forests | Critical | TD | 13 (7) | 0 (0) | 8300 |
| NT0134 | Leeward Islands moist forests | Critical | TD | 3 (0) | 8 (1) | 1000 |
| NT0155 | Puerto Rican moist forests | Vulnerable | TD | 11 (10) | 0 (0) | 7500 |
| NT0215 | Hispaniolan dry forests | Critical | TD | 20 (4) | 1 (0) | 15500 |
| NT0217 | Jalisco dry forests | Critical | TD | 17 (0) | 7 (1) | 26100 |
| NT0218 | Jamaican dry forests | Critical | TD | 1 (0) | 9 (1) | 2300 |
| NT0303 | Central American pine-oak forests | Critical | TD | 14 (2) | 19 (0) | 111400 |
| NT1306 | Cuban cactus scrub | Vulnerable | TD | 1 (0) | 18 (0) | 3300 |
| NT0107 | Caqueta moist forests | Intact | AL | 62 (2) | 8 (0) | 184200 |
| NT0112 | Central American montane forests | Vulnerable | AL | 30 (0) | 15 (0) | 13300 |
| NT0113 | Chiapas montane forests | Critical | AL | 25 (0) | 7 (1) | 5800 |
| NT0124 | Guianan Highlands moist forests | Intact | AL | 76 (14) | 12 (1) | 337600 |
| NT0125 | Guianan moist forests | Intact | AL | 98 (10) | 20 (4) | 512900 |
| NT0129 | Isthmian-Atlantic moist forests | Vulnerable | AL | 65 (5) | 27 (3) | 58900 |
| NT0137 | Magdalena-Urabé moist forests | Critical | AL | 37 (2) | 5 (0) | 76800 |
| NT0142 | Napo moist forests | Vulnerable | AL | 105 (12) | 44 (7) | 251700 |
| NT0146 | Oaxacan montane forests | Critical | AL | 26 (1) | 9 (0) | 7600 |
| NT0150 | Alto Paraná Atlantic forests | Critical | AL | 2 (0) | 10 (0) | 483800 |
| NT0174 | Ucayali moist forests | Vulnerable | AL | 83 (3) | 32 (3) | 114900 |
| NT0177 | Veracruz montane forests | Critical | AL | 15 (1) | 6 (0) | 5000 |
| NT0201 | Apure-Villavicencio dry forests | Vulnerable | AL | 0 (0) | 2 (2) | 68500 |
| NT0229 | Sin· Valley dry forests | Critical | AL | 34 (1) | 4 (0) | 25000 |
| NT0230 | Southern Pacific dry forests | Critical | AL | 42 (1) | 12 (0) | 42400 |
| NT0232 | Tumbes-Piura dry forests | Critical | AL | 8 (1) | 2 (0) | 41300 |
| NT0310 | Trans-Mexican Volcanic Belt pine-oak forests | Critical | AL | 5 (0) | 9 (0) | 91800 |
| NT0707 | Guianan savanna | Vulnerable | AL | 0 (0) | 3 (1) | 104400 |
| NT0710 | Uruguayan savanna | Critical | AL | 1 (0) | 5 (2) | 355700 |
| NT0801 | Espinal | Critical | AL | 0 (0) | 1 (1) | 108800 |
| NT0805 | Patagonian steppe | Critical | AL | 0 (0) | 7 (5) | 487200 |
| NT1001 | Central Andean dry puna | Intact | AL | 0 (0) | 8 (4) | 307400 |
| NT1002 | Central Andean puna | Vulnerable | AL | 0 (0) | 8 (1) | 161400 |
| NT1005 | Cordillera de Merida páramo | Intact | AL | 0 (0) | 2 (1) | 2800 |
| NT1008 | Southern Andean steppe | Intact | AL | 0 (0) | 3 (1) | 178200 |
| NT1316 | Tehuacán Valley matorral | Critical | AL | 14 (0) | 2 (0) | 9900 |

Priority ecoregion set (n = 66) proposed for representing all threatened Neotropical anuran species with different developmental modes (AL = aquatic larvae, TD = terrestrial development). Numbers in parentheses represent endemic species. Ecoregion conservation status obtained from [29]; threatened species combine those classified in the IUCN list as “critically endangered”, “endangered” or “vulnerable”.
