# Supplementary material for: Hung Out to Dry: Choice of Priority Ecoregions for Conserving Threatened Neotropical Anurans Depends on Life-History Traits
Source: PLoS One. 2008 May 7;3(5):e2120. doi: 10.1371/journal.pone.0002120 (PMC2361192; doi:10.1371/journal.pone.0002120)
Supplement: Table S2 — Priority ecoregions included (indicated by x) in priority sets attained with or without discriminating anuran developmental modes under different targets of species representation (90, 80 and 70%). For threatened species richness, numbers in parentheses represent endemic species. Threatened species combine those classified in the IUCN 2006 Red List as critically endangered, endangered or vulnerable. (0.12 MB DOC) [file pone.0002120.s002.doc]

Table S2. Priority ecoregions included (indicated by “x”) in priority sets attained with or without discriminating for anuran developmental modes under different targets of species representation (90%, 80%, and 70%).

| **Code** | **Ecoregion name** | **Priority** | **Threatened species richness** | | **Without discriminating anuran developmental modes** | | |  | | **Discriminating anuran developmental modes** | | |
| --- | --- | --- | --- | --- | --- | --- | --- | --- | --- | --- | --- | --- |
|  |  |  | **TD** | **AL** | **90%** | **80%** | **70%** |  | | **90%** | **80%** | **70%** |
| NT1402 | Bahamoan-Antillean Mangroves | TD, AL | 26 (2) | 1 (0) | x |  |  |  | | x |  |  |
| NT0105 | Bolivian Yungas | TD, AL | 4 (4) | 7 (6) | x | x |  | |  | x | x |  |
| NT0303 | Central American Pine-Oak Forests | TD, AL | 14 (2) | 19 (0) | x | x | x | |  | x | x | x |
| NT1003 | Central Andean Wet Puna | TD, AL | 4 (3) | 6 (5) | x |  |  | |  | x | x |  |
| NT0118 | Cordillera Oriental Montane Forests | TD, AL | 15 (4) | 12 (4) | x | x |  | |  | x | x |  |
| NT0121 | Eastern Cordillera Real Montane Forests | TD, AL | 51 (31) | 30 (17) | x | x | x | |  | x | x | x |
| NT0136 | Magdalena Valley Montane Forests | TD, AL | 31 (14) | 25 (17) | x | x | x | |  | x | x | x |
| NT1006 | Northern Andean Páramo | TD, AL | 35 (17) | 14 (7) | x | x | x | |  | x | x | x |
| NT0145 | Northwestern Andean Montane Forests | TD, AL | 63 (48) | 35 (19) | x | x | x | |  | x | x | x |
| NT0153 | Peruvian Yungas | TD, AL | 10 (7) | 20 (16) | x | x | x | |  | x | x | x |
| NT0154 | Petén-Veracruz Moist Forests | TD, AL | 13 (2) | 20 (2) | x |  |  | |  | x | x | x |
| NT0159 | Santa Marta Montane Forests | TD, AL | 4 (3) | 7 (5) | x | x |  | |  | x |  | x |
| NT0309 | Sierra Madre del Sur Pine-Oak Forests | TD, AL | 8 (1) | 18 (8) | x | x | x | |  | x | x | x |
| NT0165 | Southern Andean Yungas | TD, AL | 3 (2) | 8 (2) | x | x |  | |  | x |  |  |
| NT0167 | Talamancan Montane Forests | TD, AL | 13 (4) | 25 (11) | x | x | x | |  | x | x | x |
| NT0175 | Venezuelan Andes Montane Forests | TD, AL | 7 (6) | 24 (20) | x | x | x | |  | x | x | x |
| NT0176 | Veracruz Moist Forests | TD, AL | 8 (1) | 10 (2) | x | x |  | |  |  |  |  |
| NT0178 | Western Ecuador Moist Forests | TD, AL | 13 (2) | 9 (1) |  |  |  | |  | x |  |  |
| NT0120 | Cuban Moist Forests | TD | 23 (2) | 0 (0) | x | x | x | |  | x | x | x |
| NT0215 | Hispaniolan Dry Forests | TD | 20 (4) | 1 (0) |  |  |  | |  | x |  |  |
| NT0127 | Hispaniolan Moist Forests | TD | 36 (13) | 1 (0) | x | x | x | |  | x | x | x |
| NT0305 | Hispaniolan Pine Forests | TD | 22 (6) | 1 (0) | x |  |  | |  | x |  |  |
| NT0131 | Jamaican Moist Forests | TD | 13 (7) | 0 (0) | x | x | x | |  | x | x | x |
| NT0169 | Pantepuis | TD | 8 (7) | 2 (1) | x |  |  | |  | x | x |  |
| NT0155 | Puerto Rican Moist Forests | TD | 11 (10) | 0 (0) | x | x |  | |  | x | x |  |
| NT0150 | Alto Paraná Atlantic Forests | AL | 2 (0) | 11 (0) | x | x |  | |  | x | x | x |
| NT0201 | Apure-Villavicencio Dry Forests | AL | 0 (0) | 2 (2) |  |  |  | |  |  |  |  |
| NT0109 | Cauca Valley Montane Forests | AL | 10 (2) | 12 (7) | x |  |  | |  | x | x |  |
| NT1001 | Central Andean Dry Puna | AL | 0 (0) | 8 (4) | x |  |  | |  | x |  |  |
| NT1002 | Central Andean Puna | AL | 0 (0) | 8 (1) |  |  |  | |  | x | x | x |
| NT0115 | Chocó Darién Moist Forests | AL | 6 (2) | 13 (2) | x |  |  | |  |  |  |  |
| NT1004 | Cordillera Central Páramo | AL | 2 (1) | 5 (3) |  |  |  | |  | x |  |  |
| NT1005 | Cordillera de Mérida Páramo | AL | 0 (0) | 2 (1) |  |  |  | |  |  |  |  |
| NT0117 | Cordillera La Costa Montane Forests | AL | 3 (1) | 8 (7) | x |  |  | |  | x | x |  |
| NT0801 | Espinal | AL | 0 (0) | 1 (1) |  |  |  | |  |  |  |  |
| NT0707 | Guianan Savanna | AL | 0 (0) | 3 (1) |  |  |  | |  |  |  |  |
| NT0130 | Isthmian-Pacific Moist Forests | AL | 6 (1) | 10 (1) |  |  |  | |  |  |  |  |
| NT0805 | Patagonian Steppe | AL | 0 (0) | 7 (5) |  |  |  | |  | x | x |  |
| NT0161 | Sierra de los Tuxtlas | AL | 4 (1) | 5 (3) |  |  |  | |  | x |  |  |
| NT0308 | Sierra Madre de Oaxaca Pine-Oak Forests | AL | 6 (0) | 6 (0) |  |  |  | |  | x |  |  |
| NT1008 | Southern Andean Steppe | AL | 0 (0) | 3 (1) |  |  |  | |  |  |  |  |
| NT0310 | Trans-Mexican Volcanic Belt Pine-Oak Forests | AL | 5 (0) | 9 (0) |  |  |  | |  | x | x |  |
| NT0710 | Uruguayan Savanna | AL | 2 (0) | 5 (2) |  |  |  | |  | x |  |  |
| NT0404 | Valdivian Temperate Forests | AL | 2 (1) | 14 (12) | x | x | x | |  | x | x | x |
| **Total** |  |  | **364 (212)** | **336 (210)** | **29** | **20** | **13** | |  | **36** | **25** | **17** |

Priority sets proposed for representing threatened Neotropical anuran species with different developmental modes (AL = aquatic larvae, TD = terrestrial development). For threatened species richness, numbers in parentheses represent endemic species. Threatened species combine those classified in the IUCN 2006 Red List as “critically endangered”, “endangered” or “vulnerable”.
